# Preclinical pharmacology of a lipophenol in a mouse model of light-induced retinopathy

**DOI:** 10.1038/s12276-020-0460-7

**Published:** 2020-07-08

**Authors:** Nicolas Taveau, Aurélie Cubizolle, Laurent Guillou, Nicolas Pinquier, Espérance Moine, David Cia, Vasiliki Kalatzis, Joseph Vercauteren, Thierry Durand, Céline Crauste, Philippe Brabet

**Affiliations:** 1 Institut des Neurosciences de Montpellier, INSERM U1051, F-34091 Montpellier, France; 2 Université de Montpellier, F-34091 Montpellier, France; 3 Institut des Biomolecules Max Mousseron (IBMM), UMR 5247 - Université de Montpellier, CNRS, ENSCM, F-34095 Montpellier, France; 4 Laboratoire de Biophysique Neurosensorielle, UMR INSERM 1107, Facultés de Médecine et de Pharmacie, F-63001 Clermont-Ferrand, France

**Keywords:** Retina, Biologics, Biologics, Retina

## Abstract

Environmental light has deleterious effects on the outer retina in human retinopathies, such as *ABCA4*-related Stargardt’s disease and dry age-related macular degeneration. These effects involve carbonyl and oxidative stress, which contribute to retinal cell death and vision loss. Here, we used an albino *Abca4*^−/−^ mouse model, the outer retina of which shows susceptibility to acute photodamage, to test the protective efficacy of a new polyunsaturated fatty acid lipophenol derivative. Anatomical and functional analyses demonstrated that a single intravenous injection of isopropyl-phloroglucinol-DHA, termed IP-DHA, dose-dependently decreased light-induced photoreceptor degeneration and preserved visual sensitivity. This protective effect persisted for 3 months. IP-DHA did not affect the kinetics of the visual cycle in vivo or the activity of the RPE65 isomerase in vitro. Moreover, IP-DHA administered by oral gavage showed significant protection of photoreceptors against acute light damage. In conclusion, short-term tests in *Abca4*-deficient mice, following single-dose administration and light exposure, identify IP-DHA as a therapeutic agent for the prevention of retinal degeneration.

## Experimental & Molecular Medicine: Vision loss: Shedding light on how to prevent retinal damage

Treating retinal damage in both aging and young patients might now be easier, thanks to treatment with a lipophenol, an omega-3 fatty acid linked to an antioxidant. The retina is the part of the eye that senses light, aided by light-sensitive pigments. However, these light-sensitive pigments can be converted by light to toxic byproducts, and in some individuals, these toxic byproducts can accumulate, damaging the retina and leading to vision loss. Philippe Brabet at the Montpellier Institute of Neuroscience in France and co-workers found that lipophenol treatment protected retinal cells from damage in a mouse model of retinal disease, and that a single dose has been effective in preserving vision. These results may help in finding new treatments for retinal diseases such as Stargardt disease and age-related macular degeneration.

## Introduction

Light-induced retinal degeneration is a pathophysiological condition that affects both young and elderly people^1^. The same molecular and cellular mechanisms are often involved. In all cases, toxic photoproducts arise from vitamin A metabolites (carbonyl stressors) and oxidative stress, both of which lead to the death of outer retina cells, i.e., the retinal pigment epithelium (RPE) and photoreceptors. In young patients with autosomal recessive Stargardt’s disease (STGD1), the most common juvenile macular dystrophy, and in elderly patients with advanced aged-related macular degeneration (AMD), high levels of photoreactive metabolites in the retina enhance light-induced susceptibility^2^.

Photochemical mechanisms in the retina depend on oxygen concentration and photopigments. The high oxygen tension supplied from the dense vascular bed of the choroid exposes the outer retina to oxidation. Retinal photodamage is accompanied by lipid peroxidation^3^, suggesting that this photodamage is mediated by reactive oxygen species (ROS). Oxidative stress appears to be an early event in the retinal light damage process. There are several chromophores that absorb visible light in the outer retina, and their potential involvement in retinal photodamage was previously discussed^4^. Studies report that rhodopsin (the visual pigment) plays a critical role in photodamage^5,6^. However, the photochemical properties of rhodopsin exclude it as the photosensitizer responsible for retinal photodamage^7^. Rather, the bleaching products of rhodopsin have been suggested to be photodamage mediators^4,8^.

**Scheme 1:**
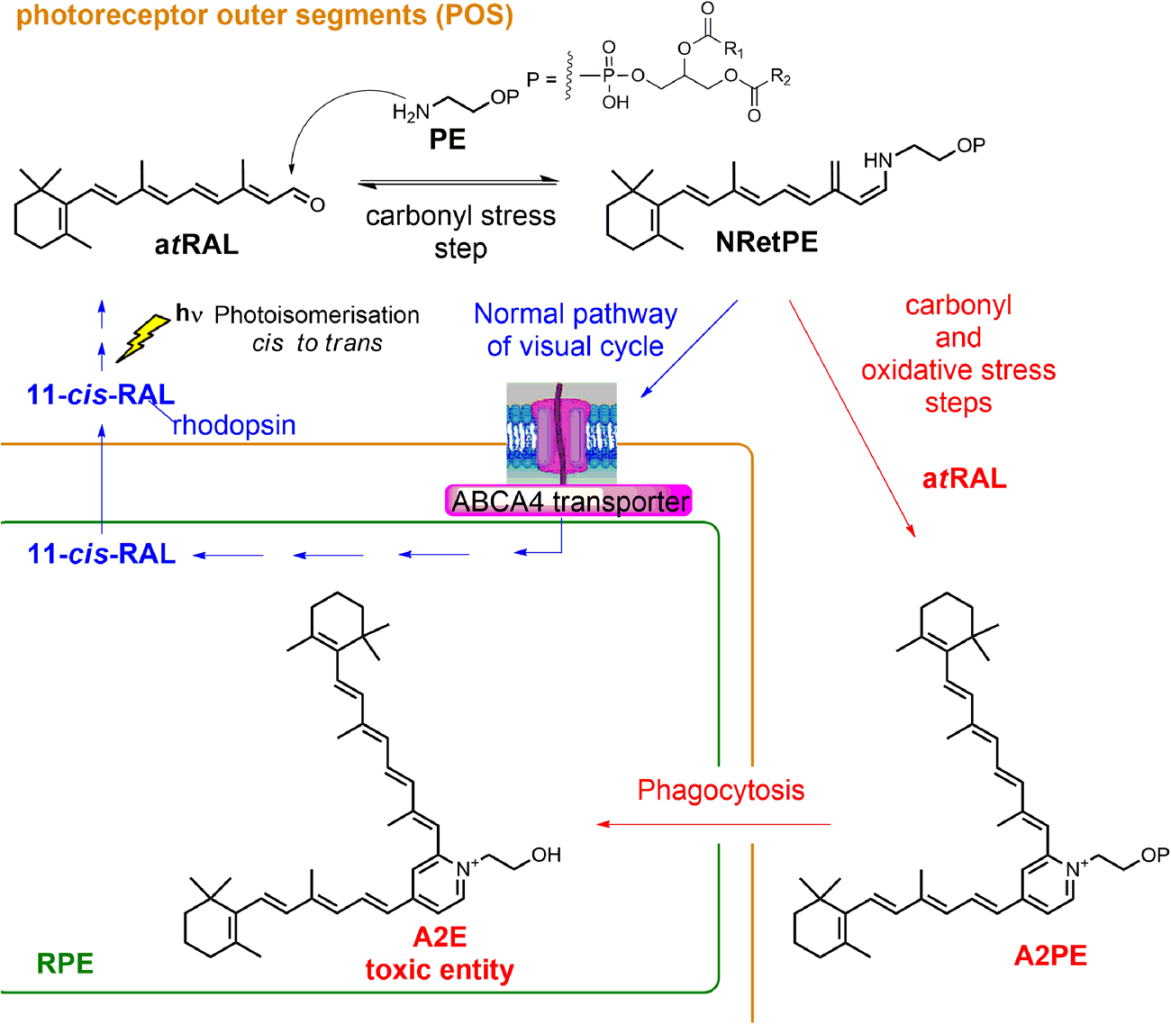
The all-trans retinal chemical pathway.

All-*trans*-retinal (a*t*RAL) is one of the products of rhodopsin photobleaching, a toxic aldehyde and a potent photosensitizer^9^. a*t*RAL forms in the photoreceptor outer segments (POS) by rhodopsin bleaching as the result of the photoisomerization of 11-*cis*-retinal (11*c*RAL), the visual pigment chromophore linked to opsin by Schiff base reaction^10^ (Scheme 1). Hydrolysis then releases a*t*RAL from the opsin protein, a*t*RAL which then transiently accumulates in the inner leaflet of the POS disc membranes^11^. The accumulation of free a*t*RAL can impose a risk of photoxidative damage to the retina induced by visible light^9^. The photodegradation of a*t*RAL can generate singlet oxygen and superoxide, which cause oxidative damage to cellular components.

Alternatively, the free a*t*RAL in the outer segments of the photoreceptors may react by condensation with phosphatidylethanolamine (PE) to form N-Retinylidene PE (NRetPE) (Scheme 1). The latter is the substrate for ABCR, the product of the ATP-binding cassette transporter A4 gene, *ABCA4*. ABCR is a flippase that facilitates NRetPE hydrolysis and a*t*RAL release^12^, which is then reduced to all-trans-retinol (a*t*ROL) by NADPH-dependent retinol dehydrogenases^13^. This reduction is the first limiting step of the visual (retinoid) cycle, a metabolic process that provides 11*c*RAL to regenerate visual pigments^14^. However, deficiency of *ABCA4*, as in the case of STGD1, strongly slows the clearance of a*t*RAL in POS and increases susceptibility of the retina to photodamage^15^. Another effect induced by the accumulation of a*t*RAL in POS is an increase in lipofuscin bis-retinoid pigments in the RPE^16^. The bis-retinoid A2PE, which arises from the condensation of two a*t*RALs with PE, following phagocytosis by RPE, generates A2E, a blue light-sensitive chromophore (430–470 nm), found in lipofuscin and toxic to the RPE^17^.

Recent reports have shown that the sequestration of a*t*RAL with primary amines or the inhibition of the visual cycle isomerase RPE65, the second limiting step of the 11*c*RAL regenerative process, can lower the a*t*RAL concentration to safe levels and prevent retinal degeneration in mutant mice^18,19^. Drugs that sequester a*t*RAL contribute to the prevention of light-induced damage^20^. The identification of new classes of sequestrating compounds would be relevant to future therapies for macular dystrophies^21^. Previously, we described the protective effect of a docosahexaenoic acid (DHA) lipophenol conjugate, IP-DHA, on ARPE-19 cells challenged with a toxic concentration of a*t*RAL^22^. More recently, we reported that IP-DHA can reduce the toxicity of a*t*RAL by acting either as an a*t*RAL or ROS scavenger or as an Nrf2-mediated enzyme defense inducer^23^, which mediates the transcription of antioxidant/cytoprotective genes^24^.

Here, we studied the therapeutic effects of IP-DHA on acute retinopathy using the *Abca4* knockout (*Abca4*^−/−^) mouse model. We demonstrate that a single intravenous injection of IP-DHA reduced the photoreceptor loss induced by light exposure and preserved visual function over time. Furthermore, IP-DHA did not affect the retinoid visual cycle. Moreover, IP-DHA administered by oral gavage using a nonoptimized formulation showed significant protection of photoreceptors.

## Materials and methods

### Chemicals

IP**-**DHA was synthetized and purified as previously described^22^. IP-DHA-a*t*RAL adducts were synthesized from isopropyl-phloroglucinol, as described in the supplemental material. DHA ethyl ester, generously gifted by Vincent Rioux (Agrocampus Ouest, Rennes, France), was purified using an n-3 polyunsaturated fatty acid (PUFA) marine oil (Omegavie 4020EE Qualitysilver, Polaris) process, as described^25^. Emixustat hydrochloride was purchased from Ambinter (Amb24183355, GreenPharma Orleans). Fatty acid-free bovine serum albumin (BSA) was purchased from Sigma-Aldrich (# A8806). A*t*RAL and all-*trans*-retinyl palmitate (a*t*RPalm) standards were purchased from Sigma-Aldrich (# R2500 and R3375, respectively), whereas 11*c*RAL was generously gifted by Rosalie Crouch (University of South Carolina, SC, USA). DPBS (1 X from Gibco, ref 14190–094) was the dilution buffer. Tropicamide (Mydriaticum 0.5% eye drops, Thea) was used to dilate the pupils, and ketamine (Imalgene 1000, Merial), xylasine (Rompun 2%, Bayer Healthcare), and cebesine (0.4% eye drops, Bausch and Lomb) were used to anesthetize the mice.

### *Abca4* knockout mice

Albino *Abca4*^+/−^ mice, bred from a mixed 129/S5 × C57BL/6J-*Tyr*^*c-Brd*^ background, were purchased from Taconic Biosciences Inc. Mice were reared and genotyped for the *Abca4* null mutation and were homozygous for Rpe65-Leu450. The lack of expression of the ABCR protein in the retina of *Abca4*^−/−^ mice was confirmed by western blot analysis (Anti-ABCA4 antibody [3F4], Novus Biologicals NBP1-30032, 1/1000; K. Damodar, personal communication). The rd8 mutant was absent in this *Abca4*^−/−^ line. Mice were subjected to standard 12-h light (90 lux) and 12-h dark cycles at a room temperature of ~22 °C and fed a standard rodent diet (SAFE 04) without DHA ad libitum. The mice were housed in facilities accredited by the French Ministry of Agriculture and Forestry (no C34-17236 – 19 December 2014). The care and use of animals was performed according to the European Directive 2010/63/EU, and the experimental procedures were approved by the French Ministry of National Education and Research (APAFIS #15117-2018051712092842v3).

### IP-DHA administration

Mice between 9 and 12 weeks of age, of both sexes, were weighed to adjust the quantity of administered compound.

For intravenous injection, the mice were divided into untreated, BSA-treated, and treated groups with IP-DHA at doses ranging from 5 to 30 mg/kg. IP-DHA was dissolved in absolute ethanol (0.1 mg/µl); the volume required for one dose of IP-DHA was mixed in sterile PBS with 0.175 g/ml lipid-free BSA in a molar ratio of 1/1–5/1 (IP-DHA/BSA). This mixture was vortexed for 2 min and incubated at 37 °C for 2 h. We assumed that IP-DHA behaves like DHA by forming complexes with BSA at the appropriate concentrations, as previously described^26^, and 100 µl was injected into the tail vein 5–10 min before exposure to light.

For oral administration, the mice were orally gavaged with a 22-gauge needle containing 100 µL of soybean oil as a control vehicle (SIO, 62053 St-Laurent-Blangy, France); emixustat (40 mg/kg) suspended in DMSO, replenished in soybean oil and containing less than 10% (v/v) DMSO; IP-DHA or DHA-ethyl ester (between 40 and 150 mg/kg) directly suspended in soybean oil.

### Light-induced retinal degeneration

After the mice were dark adapted (DA) for 24–48 h, their pupils were dilated with 0.5% tropicamide. The mice were injected with the IP-DHA/BSA complex in the dark, and then exposed to constant light (2 cool white fluorescent lamps, OSRAM of 26 watts, 1800 lm, maximum photopic efficiency of ~470 nm, light intensity averaged 24 mW/cm^2^ corresponding to ≈20.000 lux) for 2 h in a white plastic bucket with a fan. Mice were kept in the dark for 5 days before we performed final retinal evaluations. Throughout the experiment, the ambient temperature was 22 °C, identical to that of the breeding box, to avoid an increase in body temperature, which could further damage the retina^5^.

### Histological analysis

All animals were killed by cervical dislocation. The eyes were rapidly enucleated and fixed in 4% paraformaldehyde for 24 h at 4 °C. Eye cups were embedded in paraffin and cut into 5 µm sagittal sections. For hematoxylin/eosin/saffron (HES) staining, the sections were deparaffined, labeled with HES using standard protocols, rinsed and mounted in Moeviol.

### Spectral-domain optical coherence tomography (SD-OCT)

Spectral-domain optical coherence tomography (SD-OCT) was performed separately on each eye using an Envisu R2000 SD-OCT device (Bioptigen, Durham, NC). The pupils were dilated by applying 0.5% tropicamide to the cornea. The mice were anesthetized by an intraperitoneal injection of a mixture of ketamine (70 mg/kg) and xylazine (14 mg/kg), and local anesthesia was performed with a drop of oxybuprocaine (0.4% cebesine) on the cornea. Corneal hydration was maintained with Systane Ultra (Alcon, Fort Worth, TX) lubricant eye drops. Animal preparation and image acquisition were performed as previously described^27^. Analyses were performed using a rectangular scanning protocol (1.4 mm × 1.4 mm with 1000 A-scans per B-scan × 100 B-scans) while centered on the optic nerve. The thickness of the inner nuclear layer (INL), outer nuclear layer (ONL), and photoreceptor segments (PS = IS + OS) was measured and expressed in arbitrary units using ImageJ software. Twenty measurements were performed per analysis and averaged for both eyes.

### Electroretinography

All electrophysiological examinations were conducted using the Visiosystem (SIEM, France). Animals were prepared and anesthetized, and electroretinogram recording (binocular full-field ERG) was performed with cotton electrodes, as previously described^28^.

### Retinoid analysis

The mice were killed by cervical dislocation before the eyes were rapidly enucleated, frozen in liquid nitrogen, and stored at −80 °C until use. Retinoids were extracted from eyes as described^29^ and quantified from the peak areas using calibration curves determined with established standards. An A2E assay was performed on six eyes per genotype (wild type or Abca4^−/−^). Eyes were homogenized in 500 µl of absolute ethanol and centrifuged, and ethanol extracts were concentrated using a SpeedVac AES1000 (Savant Instruments, Saroo Nagar). The residual volume (~20 µl) was analyzed by reverse-phase HPLC. The absorbance of A2E and iso-A2E was measured at 430 nm, and A2E was quantified from the peak area using a calibration curve determined with synthetic A2E.

### Statistics

Statistical analyses were performed using Graph Pad Prism 5.0 software. The data of two samples were first analyzed with the Shapiro–Wilk normality test, and then two-tailed *p*-values were determined using either an unpaired Student’s *t* test or a nonparametric Mann–Whitney test. One-way ANOVA was used to compare more than two samples. Bonferroni’s or Dunn’s multicomparison adjustment was used as the post hoc test to calculate the significance levels. A *p*-value of *<* 0.05 was considered significant. The linear correlation was measured by Pearson’s *r* correlation coefficient.

## Results

### The light-induced retinal damage paradigm

**Fig. 1:**
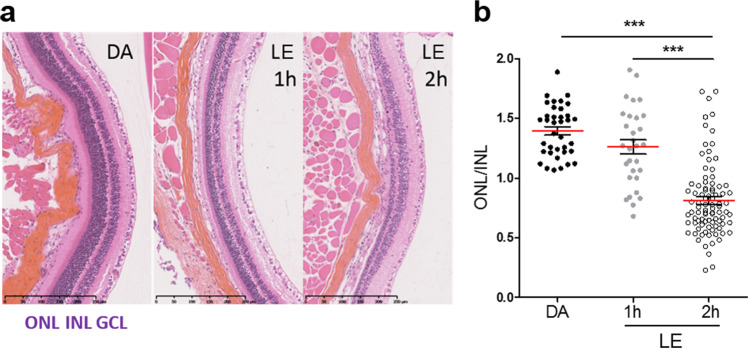
Susceptibility of retinae from *Abca4*^−/−^ mice to acute light-induced degeneration. **a** Representative hematoxylin/eosin/saffron (HES)-stained retinal sections from *Abca4*^−/−^ mice after dark adaptation (DA) or bright light exposure (LE) for 1 or 2 h followed by darkness for 5 days. ONL outer nuclear layer, INL inner nuclear layer, GCL ganglion cell layer. **b** Quantification of photoreceptor loss measured as a ratio of outer and inner nuclear layer thickness (ONL/INL) under the conditions stated in **a**. The INL thickness did not change significantly with light exposure and was used as the internal reference. Light exposure for 2 h significantly decreased ONL thickness compared to the DA control condition and 1-h light exposure. The data are presented as a scatterplot, and red bars indicate the mean ± SEM from *n* ≥ 30 ratios, ***p < 0.001 one-way ANOVA with Bonferroni’s posttest.

The albino *Abca4*^−/−^ mice, an animal model of Stargardt’s disease, used herein had a a*t*RAL level similar to that of wild-type mice in adulthood (6–7 months) but had significantly lower 11*c*RAL levels and accumulated A2E faster, according to a recent report (Supplementary Fig. S1a, S1b)^30–32^. However, there was no photoreceptor degeneration in the mice before 10 months of age (Supplementary Fig. S1c)^33^, suggesting that the level of A2E accumulation was too low to induce toxicity or that it was well tolerated in the outer retina. Therefore, to test the protective effect of IP-DHA on photoreceptor cells, we used a paradigm of acute retinal damage induced by light in mice aged 9–12 weeks (see Material and methods section). These mice were reared in darkness for 24–48 h, an appropriate amount of time for regenerating the visual pigment (opsin + 11*c*RAL), and then exposed to bright light. The overproduction and photooxidation of a*t*RAL caused by intense light illumination and the genetic defect in the *Abca4* gene can induce delayed photoreceptor degeneration, which was quantified after 5 days in the dark by retinal histological analysis (Fig. 1). Light exposure for 2 h produced a reduction in the thickness of the photoreceptor ONL, which was 58.0 ± 0.2% of that of mice not exposed to light. Exposure for 1 h caused only a 10.0 ± 0.4% reduction. The ONL/INL ratio represents the OCT results with the thickness of the INL used as an internal standard as it does not significantly change after light exposure. All protective assessments were therefore conducted after a 2-h light exposure.

### IP-DHA treatment prevents light-induced acute retinal degeneration

**Fig. 2:**
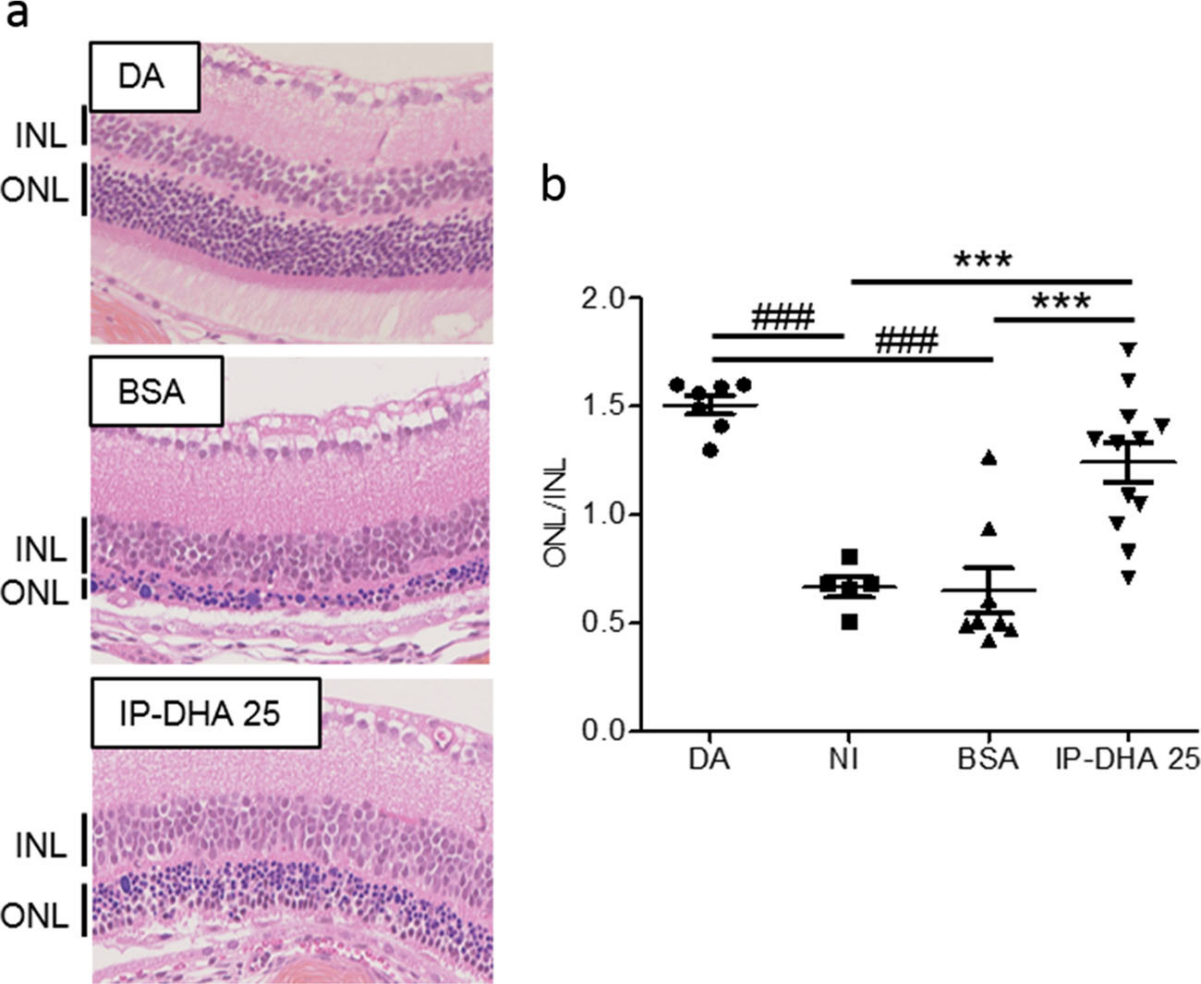
Histological analysis of retinae after light-induced acute retinal degeneration. **a** HES staining of retinal sections from *Abca4*^−/−^ mice that were dark-adapted (DA) or injected with BSA (BSA) or 25 mg/kg IP-DHA (IP-DHA 25) before light exposure. **b** IP-DHA treatment prevented the thinning of the outer nuclear layer (ONL), which contains photoreceptors, in untreated or BSA-treated mice. The data are presented as scatterplots, and horizontal bars indicate the mean ± SEM from *n* ≥ 6 mice. One-way ANOVA with Bonferroni’s test: ^###^*p* < 0.001 vs. DA; ***p < 0.001 vs IP-DHA.

**Fig. 3:**
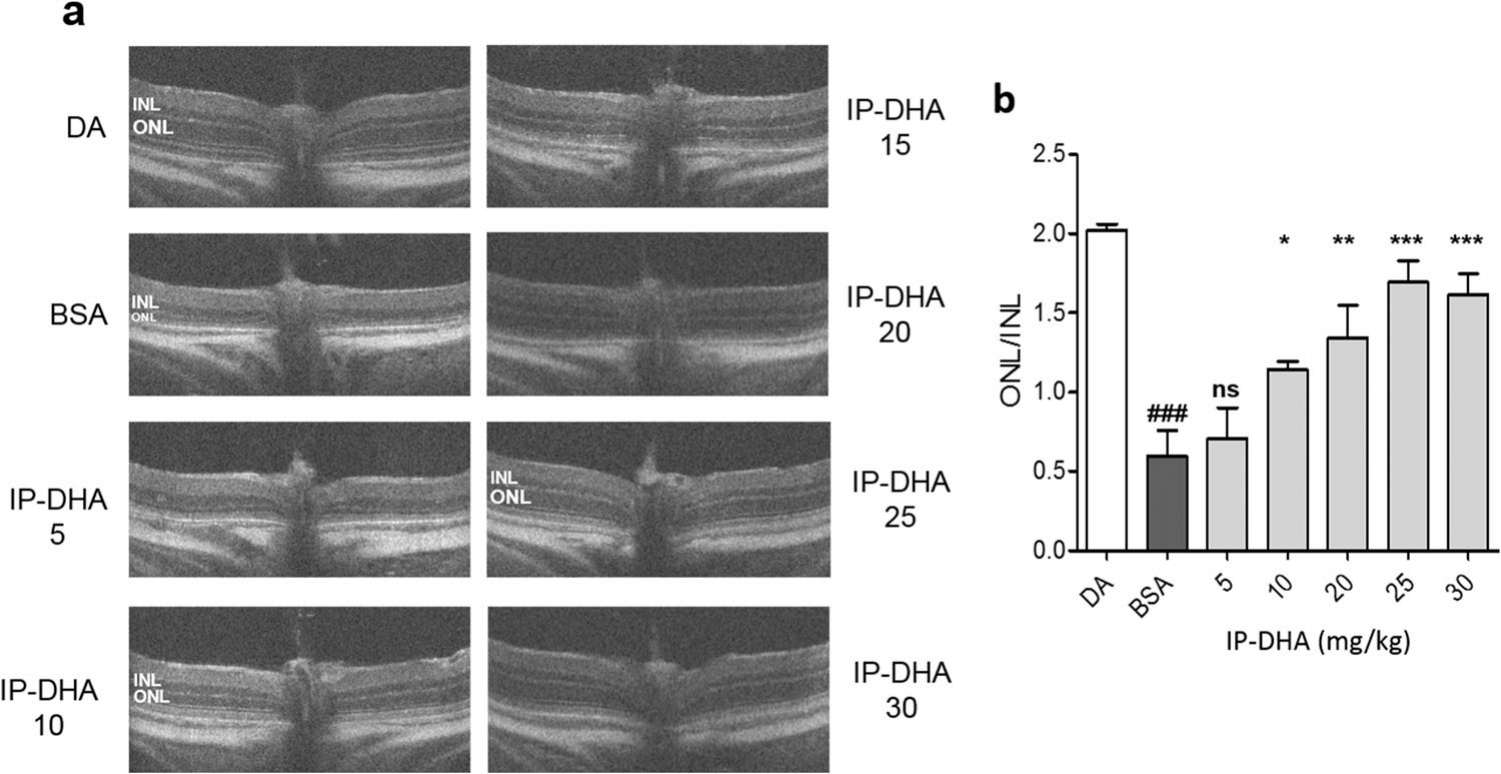
SD-OCT imaging after light-induced acute retinal degeneration. **a** SD-OCT images show reduced thickness of the ONL 5 days after light exposure in *Abca4*^−/−^ mice administered BSA (vehicle), whereas those receiving 25 mg/kg IP-DHA (IP-DHA 25) exhibited an ONL thickness similar to that of nonexposed mice (DA). **b** The protective effect of IP-DHA was dose dependent, with significant differences observed with doses ≥10 mg/kg. *Abca4*^−/−^ mice treated with 25 mg/kg IP-DHA recovered 80–85% of ONL thickness, whereas no protection was observed in BSA-treated mice (BSA). The data are represented as the mean ± SEM from *n* ≥ 5 mice. One-way ANOVA with Bonferroni’s multicomparison posttest: ^###^*p* < 0.001 BSA vs. DA; *p < 0.05, **p < 0.01, ***p < 0.001 IP-DHA vs. BSA; IP-DHA 5 vs. BSA nonsignificant, ns.

**Fig. 4:**
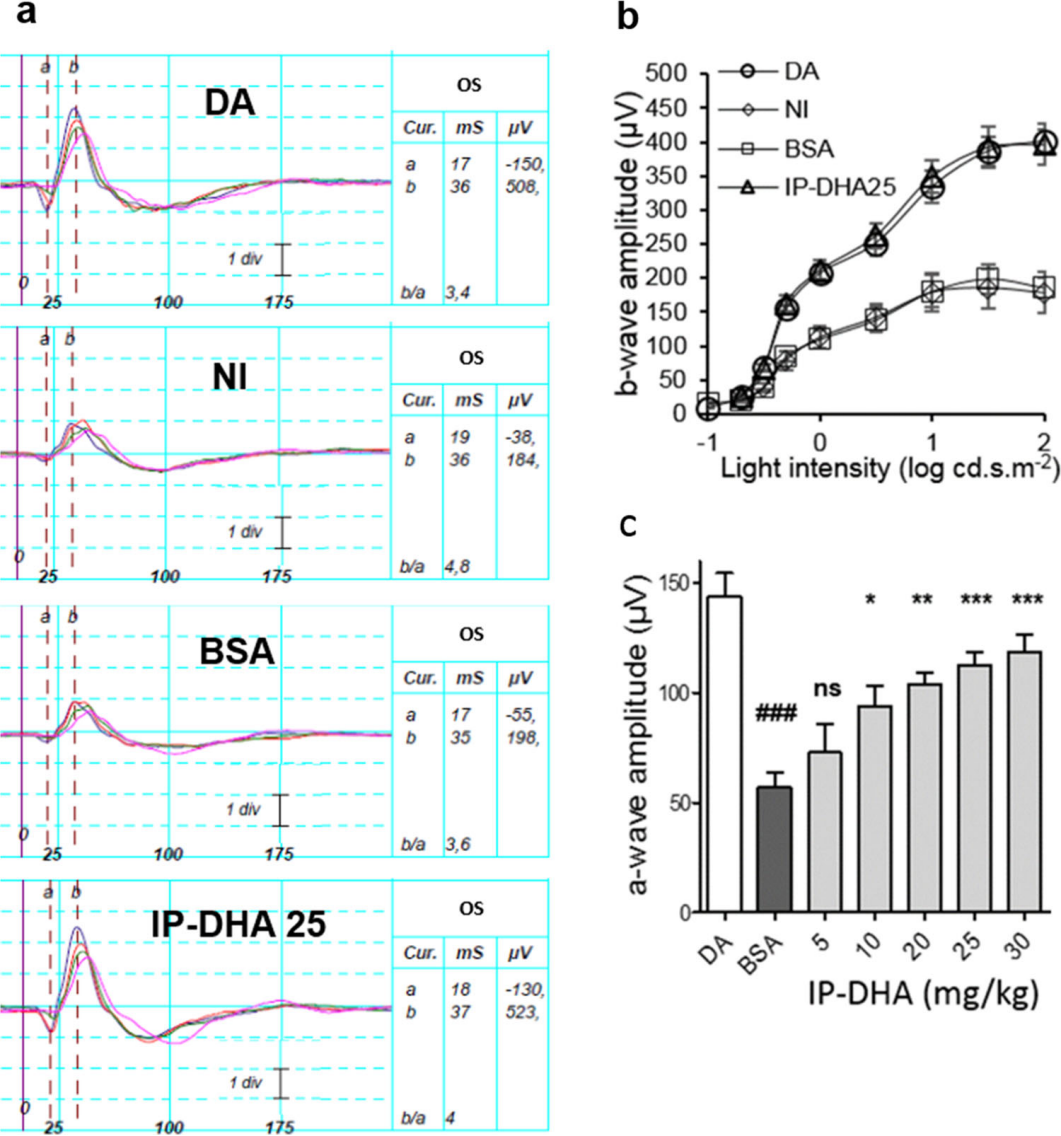
Full-field ERG recordings after light-induced acute retinal degeneration. **a** Full-field ERG responses of IP-DHA (25 mg/kg)-treated or BSA-treated *Abca4*^−/−^ mice following light exposure were recorded under scotopic conditions and compared to nonexposed (DA) and noninjected (NI) light-exposed littermates. Data are representative ERG responses from left eyes (OS) to a light intensity of 0 to 2-log cd.s.m^−2^. Amplitude scale, 1 div = 156 µV. **b** ERG b-wave amplitudes plotted as a function of light intensity were fully preserved in IP-DHA 25-treated *Abca4*^−/−^ mice compared to BSA-treated animals. Error bars indicate the SEM of the means (*n* ≥ 5). **c** The dose-dependent protection of ERG a-wave amplitudes by IP-DHA at 2-log cd.s.m^−2^. Error bars indicate the SEM (*n* ≥ 5). One-way ANOVA with Bonferroni’s multiple comparison posttest: ^###^*p* < 0.001 BSA vs. DA mice; *p < 0.05, **p < 0.01, ***p < 0.001 IP-DHA versus BSA-treated mice; BSA vs. IP-DHA 5 nonsignificant, ns.

**Fig. 5:**
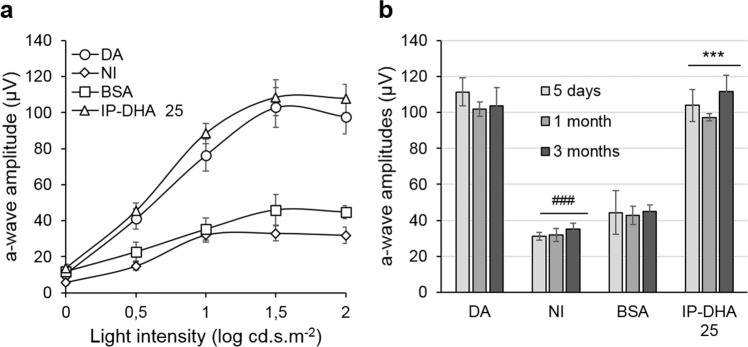
Effect of IP-DHA on light-induced degeneration over time. **a** Full-field ERG responses of *Abca4*^−/−^ mice 3 months after a single IP-DHA injection. ERG scotopic responses were recorded, and a-wave amplitudes were plotted as a function of light intensity. IP-DHA (25 mg/kg)-treated mice exposed to light showed an a-wave amplitude similar to that of DA control mice, whereas BSA-treated and untreated (NI) mice exposed to light did not recover amplitudes. The data are presented as the mean ± SEM from *n* = 5–6 mice. **b** a-wave amplitudes recorded at 2-log cd.s.m^−2^ did not change in each treatment over the 3-month period. The data are presented as the mean ± SEM from *n* = 5 mice. One-way ANOVA with Bonferroni’s multiple comparison posttest: ^###^*p* < 0.001, NI vs. DA; ***p < 0.001, IP-DHA25 mg/kg vs. BSA; IP-DHA vs. DA, nonsignificant.

IP-DHA is an oily compound that is poorly soluble in water because of its long PUFA chain (DHA). To test its effect on acute light-induced damage, IP-DHA was complexed to defatted BSA and administered in the dark by intravenous injection in the tail vein as a single dose (5–30 mg/kg) prior to light exposure. Five days post-light exposure, the mice were evaluated by in vivo retinal imaging by histological analysis (Fig. 2), spectral-domain optical coherence tomography (SD-OCT, Fig. 3), and full-field electroretinography (ERG, Fig. 4). Whereas BSA injection did not prevent retinal degeneration and induced an average loss of photoreceptors comparable to no injection (57.9 ± 6.8% and 56.4 ± 2.5%, respectively, BSA vs NI, Fig. 2b), IP-DHA prevented light-induced acute degeneration and enabled the recovery of 80–85% of intact photoreceptors in a dose-dependent manner (Figs. 2b and 3b). IP-DHA injection markedly rescued the b-wave and a-wave amplitudes (Fig. 4a, b). A strong correlation between anatomical (ONL/INL) and functional (ERG) measurements suggested that the conservation of light-detecting photoreceptor cells was the cause of visual sensitivity rescue (Fig. S2). The level of 11*c*RAL was also well correlated with the a-wave amplitude, making 11*c*RAL a biomarker of photoreceptor status. The rescue of photoreceptor function (a-wave amplitude) lasted for at least 3 months postinjection (Fig. 5). By contrast, the visual function of untreated and BSA-treated mice was unchanged, indicating a permanent deficit that lasted more than 3 months after light exposure. During these 3 months, the mice showed normal weight gains and were generally indistinguishable from each other. Overall, this posttreatment follow-up does not suggest any adverse dose-related effects. Collectively, these results emphasize the efficacy of IP-DHA in preventing acute retinal damage and support the protective effect of therapy with this compound.

### Protection in mice does not affect the retinoid cycle

**Fig. 6:**
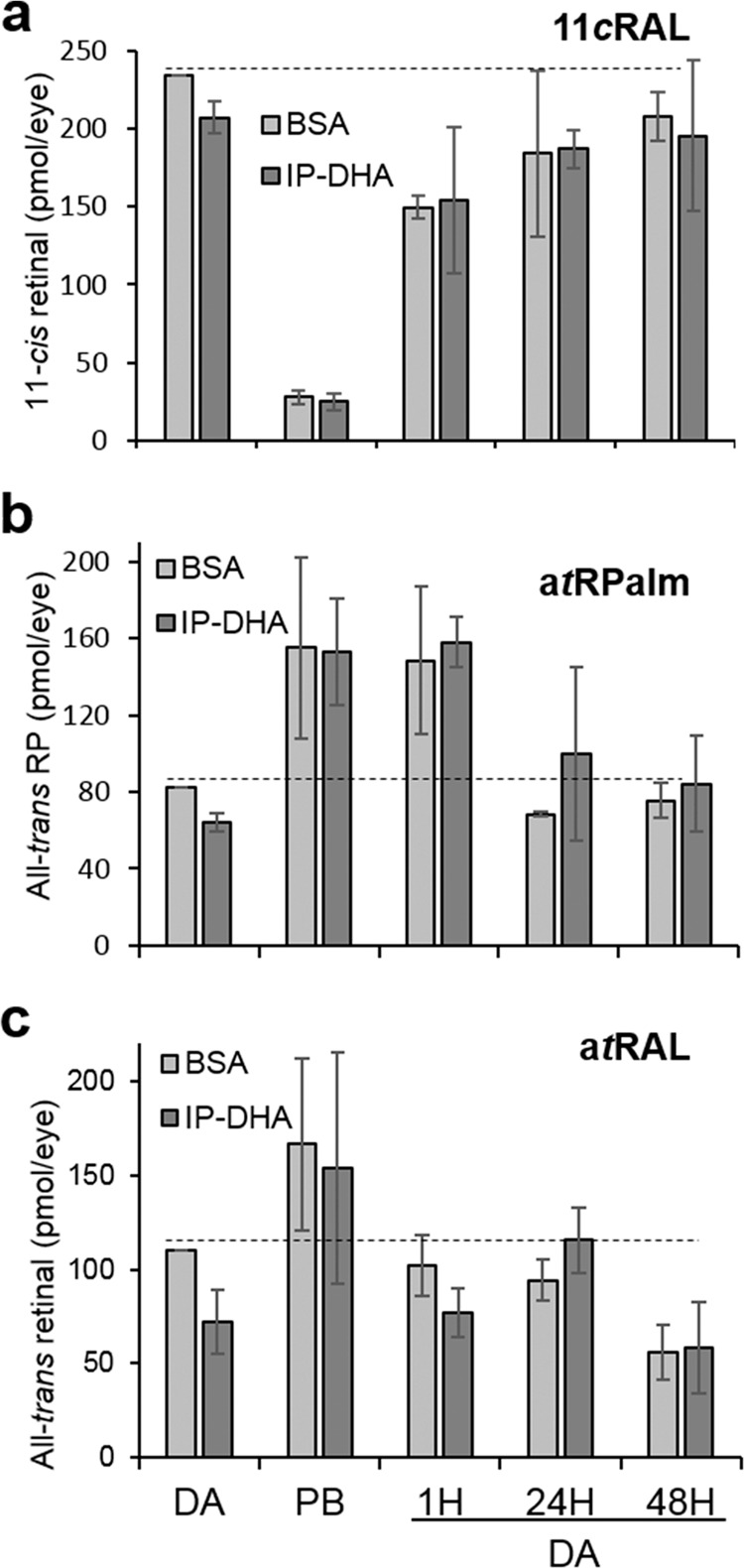
Regeneration kinetics after short light exposure. Regeneration kinetics of 11*c*RAL **a**, a*t*RPalm **b**, and a*t*RAL **c** were examined after ~90% rhodopsin bleaching by illumination at 24 mW/cm^2^ for 2 min. *Abca*4^−/−^ mice kept in the dark for 24 h (DA) were administered BSA or BSA + IP-DHA (25 mg/kg) in the tail vein 5 min before the 2-min light exposure. Prior to (DA) or after bleaching (PB), the mice were returned to the dark for the indicated time (DA 1H, 24H, and 48H), and retinoid quantification was subsequently performed by normal-phase HPLC. Error bars represent the SD, *n* = 3.

We asked whether IP-DHA might react in vivo with a*t*RAL and impair chromophore regeneration. By exposing the mice to bright light for 2 min, a*t*RAL reached maximum levels prior to its reduction (Supplementary Fig. S3a). We then returned the mice to the dark for several hours before HPLC analysis of retinoid levels (Figs. S3b and 6). Chromophore 11*c*RAL levels decreased by ~90% after bleaching and then regenerated within the first hour of dark adaptation, whereas a*t*RPalm accumulated postbleaching and did not return to baseline levels until 24 h postadaptation (Fig. 6a, b). By contrast, a*t*RAL increased after bleaching and declined rapidly to basal levels after 1 h of dark adaptation (Fig. 6c). IP-DHA tended to reduce a*t*RAL in the dark before and after bleaching, but this effect was not significant. We conclude that IP-DHA might partially reduce a*t*RAL without affecting the retinoid cycle.

### Comparative effects of IP-DHA and emixustat by oral pretreatment

**Fig. 7:**
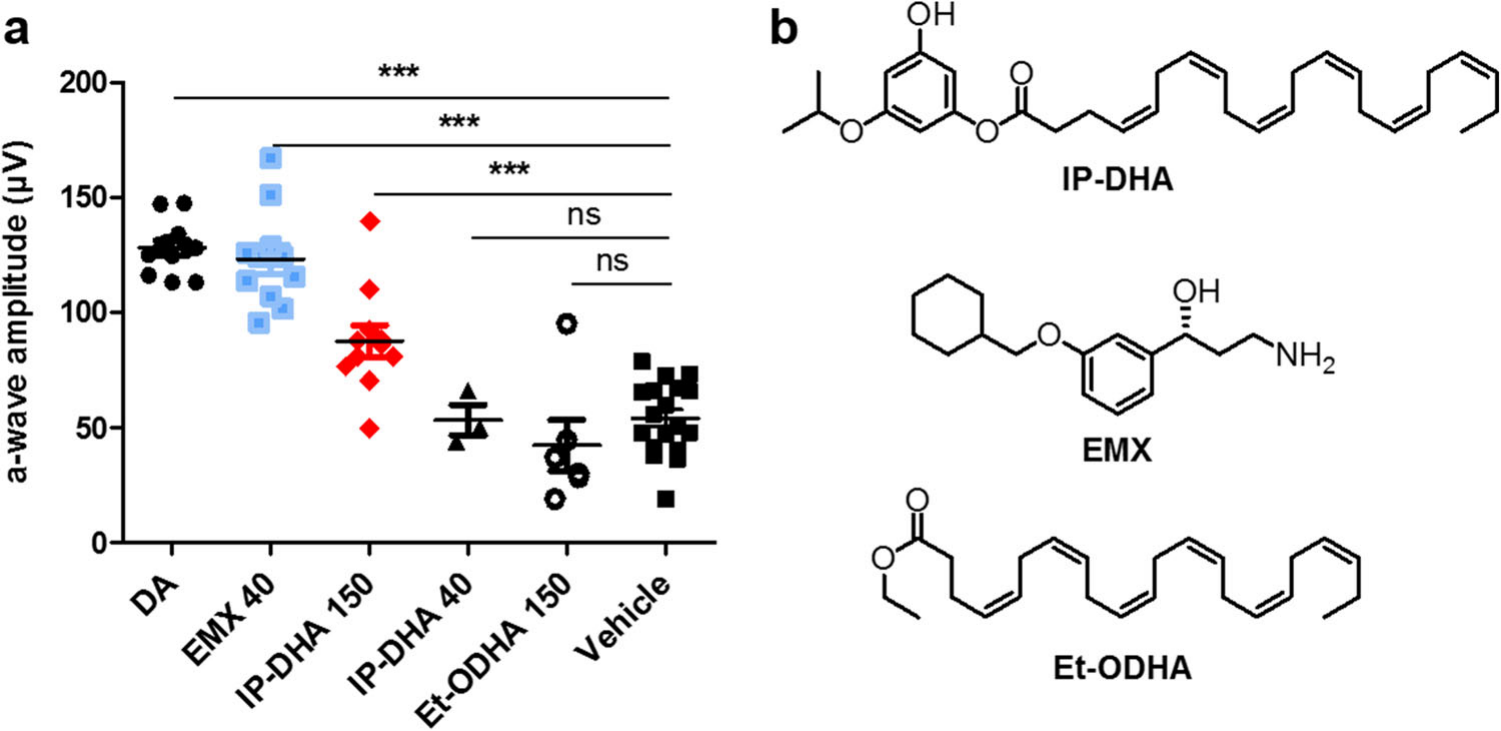
Comparative protective effects of IP-DHA, DHA ethyl ester, and EMX on light-induced retinal degeneration. **a** Dark-adapted (DA) 8–9-week-old *Abca*4^−/−^ mice were treated by oral gavage with emixustat (EMX, 40 mg/kg, 24 h), IP-DHA (40 and 150 mg/kg, 6 h), or DHA ethyl ester (Et-ODHA 150 mg/kg, 6 h), pupils were dilated, and mice were exposed to light for 2 h. The mice were returned to the dark for 5 days before full-field ERG recordings. The a-wave amplitudes were recorded at 2-log cd.s/m^2^ light intensity for each treatment and statistically compared to the amplitude of mice treated with the vehicle (soybean oil). Data are expressed as the mean ± SEM. Three asterisk, one-way ANOVA with Bonferroni’s multicomparison test: *p* < 0.001, DA (*n* = 12), 40 mg/kg EMX (*n* = 11), 150 mg/kg IP-DHA (*n* = 11), 40 mg/kg IP-DHA (*n* = 3), 150 mg/kg Et-ODHA (*n* = 6) vs. soybean oil (oil, *n* = 18); ns nonsignificant. **b** Chemical structures of IP-DHA, EMX, and Et-ODHA.

A comparison of IP-DHA with emixustat (EMX), well known to have protective effects against acute light-induced retinal degeneration^19^, showed that these treatments had similar effects (Fig. 7). Both compounds generated higher a-wave amplitudes in the rod and cone ERG responses than did the oil vehicle (*p* < 0.001). EMX treatment (40 mg/kg) by oral gavage 24 h before light exposure protected more than 90% of the photoreceptor response, which was not significantly different from the photoreceptor response of unexposed mice (DA). IP-DHA showed a dose-dependent effect after 6 h of pretreatment, protecting up to 70% of the photoreceptor ERG response at the highest dose (150 mg/kg). Thus, even in the absence of an optimized oral formulation, IP-DHA had a protective effect against light-induced retinal degeneration, which was promising. Remarkably, DHA ethyl ester (Et-ODHA, 150 mg/kg) had no protective effect, suggesting that simple DHA supplementation is not sufficient to provide photoreceptor protection and thus that the isopropyl-phloroglucinol moiety is a necessary part of the lipophenol structure to observe IP-DHA activity.

### Effect of emixustat and IP-DHA on RPE65 isomerase

**Fig. 8:**
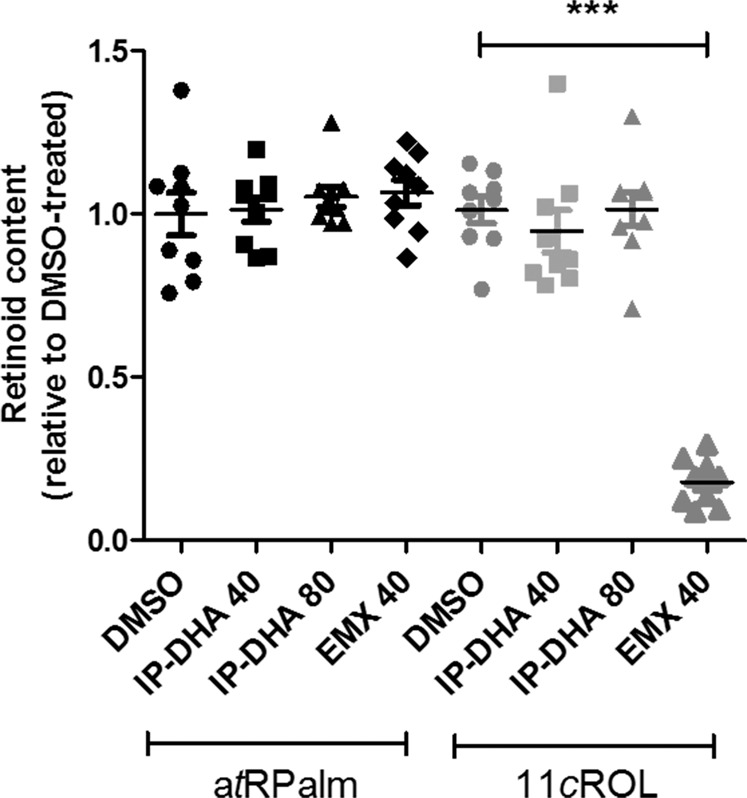
Emixustat, but not IP-DHA, inhibits RPE65 isomerase activity. HEK-LR cells stably expressing LRAT-CFP and RPE65-GFP were plated in T25 flasks at 10^5^ cells/ml and grown for 24 h before DMSO (CTL), IP-DHA (40 and 80 µM), or EMX (40 µM) was added. Cells were cultured for 24 h and incubated with 10 µM a*t*ROL for the last 24 h. Normal-phase HPLC was used to analyze the retinoid extracts from the cells. Data are presented as the mean ± SEM. ***n = 3 independent experiments with each treatment performed in triplicate. One-way ANOVA Kruskal–Wallis test with Dunn’s multiple comparison test: *p* < 0.001 EMX vs. DMSO. Black dots represent LRAT activity, gray dots represent RPE65 isomerase activity.

EMX is a visual cycle modulator that inhibits the RPE65 isomerase of the visual cycle, slows the regeneration of 11*c*RAL and thus limits the rate of a*t*RAL produced by light^34^. A first explanation for the difference in the protective efficacies of EMX and IP-DHA may be the inhibition of RPE65. We thus tested whether we could reproduce the inhibitory effect of EMX in HEK cells expressing LRAT and RPE65 (HEK-LR) and compared the inhibitory effect of EMX to that of IP-DHA in this model. HEK-LR cells were incubated initially with 40 µM EMX or with 40–80 µM IP-DHA for 24 h and then with 10 µM vitamin A for an additional 24 h. Retinoids were extracted from cell lysates and analyzed by normal-phase HPLC. As shown in Fig. 8, neither IP-DHA nor EMX affected the production of a*t*RPalm catalyzed by LRAT, and only EMX significantly inhibited the formation of 11*c*ROL catalyzed by RPE65. Therefore, our data show that IP-DHA does not affect RPE65 isomerase in vitro, unlike EMX.

## Discussion

In the present study, we evaluated the therapeutic efficacy of a new small molecule, a phloroglucinol derivative, referred to as lipophenol (or phenolipid). The isopropyl function of this molecule reinforces the nucleophilic nature of its aromatic ring, and DHA should improve its bioavailability and its capacity to reach the outer retina. Our previous in vitro evaluation of cultured retinal cells has shown that this lipophenol reduces the toxicity of a*t*RAL (anti-carbonyl stress activity) and activates redox enzymes to promote antioxidant defenses^23^. We continued this preclinical evaluation with an in vivo study in mice using a light-induced photoreceptor degeneration model in the context of an *Abca4* gene deletion that mimics human STGD1. Here, we will discuss the relevance of the mouse model as well as the structure-function relationship of lipophenol, its mode of action, and its comparison with small molecules already used in the treatment of macular degeneration.

### Acute light-induced retinal degeneration in Abca4^−/−^ mice

First, we examined the retinoid composition of the eyes of adult *Abca4*^−/−^ mice (Supplemental Fig. S1a). These mice showed no accumulation of a*t*RAL when subjected to standard light cycles, a result consistent with the recent report by Adler and colleagues^32^. By contrast, 11*c*RAL levels were lower in *Abca4*^−/−^ mice than in wild-type mice, suggesting the slower regeneration of chromophores. An explanation of this finding is that A2E accumulates faster over time in the *Abca4*^−/−^ RPE than in the wild-type RPE (Supplemental Fig. S1b), a process that involves a delay in the removal of a*t*RAL and its condensation with PE, rather than its entry into the visual retinoid cycle to regenerate 11*c*RAL. Moiseyev et al.^30^ also described that A2E inhibits the regeneration of 11*c*RAL by binding to the RPE65 isomerase. Therefore, the lower rate of 11*c*RAL production is probably due to a defect in the two limiting steps of the visual cycle, consecutively, the reduction of a*t*RAL to a*t*ROL facilitated by ABCR at the POS and the *trans-cis* isomerization of retinol catalyzed by RPE65 in the RPE. As we did not observe a significant decrease in scotopic photosensitivity before 10 months of age (Supplemental Fig. S1c), which corresponds to the OCT measurements reported previously^33^; younger Abca4^−/−^ mice do not exhibit retinal degeneration without exposure to intense light. This observation highlights the difference between the late onset of *Abca4*-related retinal degeneration in mice and the early adolescent onset of macular degeneration in humans. This difference is related to human retinal features, such as the presence of a macula and a much lower photoreceptor cell density^35^. This is a limitation of the mouse model that can be overcome experimentally by exposure to light. Moreover, in patients with foveal sparing, vision loss was not noticed until adulthood^36^, a clinical characteristic that, in association with lipofuscin accumulation, may be similar to the mouse phenotype^37,38^.

We also demonstrated here that the acute form of light-induced retinal degeneration in *Abca4*^−/−^ mice provides a good opportunity to reconstruct the temporal sequence of events leading to cell death. A prevalent finding, observed by SD-OCT imaging, was hyperreflectivity in the ONL at the earliest stages of retinal damage (up to 1 day postbleaching) without thinning of the retinal outer layer (Supplemental Fig. S4). Recently, hyperreflectivity in the outer nuclear layer of Stargardt patients was documented by OCT^39^. The authors suggested that hyperreflectivity is the result of pathological alteration of the ONL, mainly in the cones. In this study, bright light damaged both the rods and cones of *Abca4*^−/−^ mice (Supplementary Fig. S5). In the genetic context of *Abca4* deficiency, light-induced retinal lesions appear to be an accelerated pathophysiological condition, which best represents STGD1 disease in mice.

### Potency of lipophenol IP-DHA to protect photoreceptors from light-induced damage

Polyphenols have long been recognized as antioxidants; more recently, some of them have been shown to have anti-carbonyl stress activities, and their application in the treatment of neurodegenerative diseases has been widely highlighted in the past few years^40^. Among polyphenols, phloroglucinol is a monomer of phlorotannin with anti-carbonyl and oxidative stress (anti-COS) activities, which also displays therapeutic potential for neurodegenerative diseases^41^. We previously reported the in vitro protective effects of phloroglucinol in outer retinal cells by reducing oxidative status and trapping a*t*RAL^42^. However, a major disadvantage of phloroglucinol is its low bioavailability in the retina (unpublished data). Our strategy to improve selectivity for the retina relied on chemical modifications of the polyphenol core, leading to additional lipophilic derivatives. We synthesized phloroglucinol derivatives with DHA (omega-3, C22:6, n-3) linked on a phenolic functional group. The choice of DHA was dictated by (1) its high content in the photoreceptor disc membrane, the photoisomerization site that produces a*t*RAL, and (2) several benefits in the retina that were previously described^43–46^. The second modification performed on phloroglucinol was the introduction of an isopropyl functional group, whose electron-donating inductive effect should adjust the nucleophilicity of the aromatic ring to trap a*t*RAL most efficiently.

With regard to these data, we evaluated the effect of IP-DHA on acute light-induced retinal degeneration in 9–12-week-old mice, which required only a 1-week experimental procedure. Similar experimental paradigms were extensively reported for testing the effects of small molecules on the development of acute light-induced retinal damage in mutant mice^18,34,47–50^. We show that the use of IP-DHA as a therapeutic agent is very effective in protecting the outer retina of *Abca4*^−/−^ mice from light-induced damage. This protective effect was dose-dependent and prevented both photoreceptor cell death and photosensitivity. The morphological (ONL/INL ratio in OCT and histological sections) and functional (a- and b-wave ERG) measurements were well correlated, suggesting a direct relationship between the number of surviving photoreceptors and the sensitivity of the retina to light stimulation. These changes also occur in the inner and outer segments of the photoreceptors, with a strong correlation of their lengths with the thickness of the nuclear layer and the light response (Supplemental Fig. S6). Together, these findings support that IP-DHA acts at the level of photoreceptor cells to maintain their integrity by preventing toxicity from the accumulation of free a*t*RAL, which is produced by exposure to light in *Abca4*^−/−^ mice.

### What is the mechanism of action of IP-DHA?

A major question is the mechanism of action by which IP-DHA reduces the toxicity of a*t*RAL. Phloroglucinol and isopropyl-phloroglucinol form irreversible chromene adducts with a*t*RAL^42^ (Supplementary Figs. S7 and S8), suggesting that the isopropyl moiety can contribute to the reactivity of phloroglucinol. However, no adduct was identified or isolated under the same conditions by mixing IP-DHA and a*t*RAL in test tubes. We chemically synthesized four potential adducts that could result from an IP-DHA reaction with atRAL (Supplemental Figs. S7 and S8). First, we did not detect IP-DHA-a*t*RAL synthetized adducts in cell extracts after coincubation of IP-DHA and atRAL with ARPE-19 cells or in the retina after IP-DHA IV administration. Second, we were unable to identify other adducts structurally compatible with the reaction of IP-DHA on a*t*RAL. This result can be explained either by the instability of the IP-DHA-atRAL adducts (which would have different chemical structures) or by the low reactivity of IP-DHA with atRAL owing to the presence of DHA. We recently reported that several enzyme systems responsible for detoxifying ROS in the cell (antioxidant/electrophile response element (ARE/EpRE)-activated enzymes, regulated by the Nrf2/Keap1 pathway^24^) are activated by IP-DHA^23^. Now, we assume that the clearance of ROS is accomplished mainly through this mechanism. Among the enzymes activated by Nrf2 pathways, some enzymes target ROS but are also able to detoxify aldehyde derivatives, such as glutathione S-transferase, which also catalyzes the formation of the glutathione–aldehyde adduct (with HNE, for instance)^51^. In addition, other enzyme systems could detoxify the retina, such as dehydrogenases. For example, ALDH1 and ALDH2 are described as enzymes involved in aldehyde detoxification^51^ and that use atRAL as a substrate^52^. Overall, these data do not support the concept of the direct trapping of atRAL by IP-DHA in its lipophenolic form but rather a reduction in atRAL toxicity by acting on enzymatic cellular defenses capable of reducing carbonyl and oxidative stress. A final mode of action may be the release of phloroglucinol and the DHA moiety by enzymatic cleavage of lipophenol, both of which have a separate protective effect. However, no protective effect of DHA ethyl ester is shown here, suggesting that supplementation with DHA alone is not sufficient to ensure photoreceptor protection.

### What benefit can lipophenol provide against STGD1 and dry AMD compared to other evaluated molecules?

Therapeutic agents target visual cycle inhibition and thereby reduce a*t*RAL formation^20,21,53^. Small molecules may slow down the visual cycle by reducing the amount of circulating vitamin A (fenretinide and A1120 RBP4 antagonist) or by inhibiting the isomerase activity of RPE65 (isotretinoin, retinylamine, and EMX). A*t*RAL sequestrators (primary amines, VM-200, and EMX) and the tri-deuterated form of vitamin A (ALK-001) were able to slow the formation of bis-retinoids^54^. The visual cycle modulator EMX is a primary amine that traps aldehyde in the photoreceptor, binds to and inhibits RPE65 in RPE^19^. Recently, the most advanced oral treatment with EMX failed to reduce geographic atrophy in phase 2b/3 trials^55^. The adverse events were likely related to the drug’s mechanism of action, RPE65 inhibition leading to decreased availability of 11-*cis*-retinal to the photoreceptors. New therapies that do not directly target visual cycle inhibition would be required to prevent severe adverse effects. Thus, the use of IP-DHA might be more beneficial than that of primary amines, as IP-DHA can act both on retinal toxicity and on the production of reactive carbonyl and oxygen species involved in the death of RPE and photoreceptors. The kinetics of chromophore regeneration after IP-DHA treatment (Fig. 7) revealed that IP-DHA does not impair the visual cycle. In addition, IP-DHA does not inhibit RPE65 isomerase; hence, it should not present side effects arising from this inhibitory outcome. Regarding toxicity, most primary amines are much more nucleophilic than the aromatic lipophenol; thus, they should present more potential toxicity than lipophenol. Furthermore, two natural components compose lipophenol: one polyphenolic moiety, an antioxidant compound present in foods, named phloroglucinol, an antispasmodic compound already approved by the FDA (Spasfon®), and DHA, a natural PUFA omega-3 present in food and beneficial for vision. We previously showed that the addition of isopropyl did not amend the low toxicity level of lipophenols^23^. For all of these reasons, the risk of toxicity of lipophenols may be considered low. Validating this assumption, after a single injection, we observed no toxicity for over 3 months. However, to exclude all risks, future chronic treatment analyses should include toxicological studies.

In summary, based on the in vitro and in vivo biological activities of the main investigated molecule, IP- DHA, anti-COS lipophenols could represent a novel class of compounds that prevent retinal degeneration. Currently, several clinical trials on STGD1 are ongoing (https://www.clinicaltrials.gov/)^56,57^. Two molecules that act on the visual cycle are in clinical phase 2/3 development (ALK001, EMX). Concomitant nonpharmacologic approaches, such as stem cell (ClinicalTrials.gov NCT02445612) or gene delivery therapies (ClinicalTrials.gov NCT01736592), and pharmacologic treatments may be the key for efficient bitherapy. The therapeutic window for pharmacological treatment may be broader than that for other treatments if the compound shows preventive effects and is able to slow down the progression of the disease. Our approach is novel because lipophenols are able to target both carbonyl (a*t*RAL) and oxidative stresses. This double reactivity represents a novel aspect of the therapeutic action of phenolic derivatives and addresses a challenge in the treatment of macular degeneration.

## Supplementary information

Figures S1 to S2

## Patent

Brabet, P., Cia, D., Guillou, L., Hamel, C., Vigor, C., Durand, T., Crauste, C., Vercauteren, J. New lipophenol compounds and uses thereof, WO2015162265A1 (2015).
