# Supplementary material for: Preclinical pharmacology of a lipophenol in a mouse model of light-induced retinopathy
Source: Exp Mol Med. 2020 Jul 8;52(7):1090–101. doi: 10.1038/s12276-020-0460-7 (PMC8080701; doi:10.1038/s12276-020-0460-7)
Supplement: Supplementary file 1 — Figures S1 to S2 [file 12276_2020_460_MOESM1_ESM.docx]

**Preclinical pharmacology of a lipophenol in a mouse model of light-induced retinopathy**

*Nicolas Taveau ^1^***, Aurélie Cubizolle ^1^***, Laurent Guillou ^1^, Nicolas Pinquier* ^1^*, Espérance Moine ^2^, David Cia ^3^,* Vasiliki Kalatzis^1^, *Joseph Vercauteren ^2^, Thierry Durand ^2^, Céline Crauste ^2^*^®^*, Philippe Brabet ^1^*^®^*^#^.*

**Supplementary information**

**Figure S1.** Quantification of retinal, A2E, and full-field ERG in wild type and Abca4^-/-^ mice.

**Figure S2.** Correlation between anatomical and functional evaluations

**Figure S3.** Kinetics of retinoid in bleached Abca4^+/-^ mice

**Figure S4.** Kinetic of retinal damage in darkness after acute light exposure

**Figure S5.** The light responses of rod and cone photoreceptors

**Figure S6.** Correlation between photoreceptor segment length, outer nuclear layer and photoresponse.

**Figure S7.** Chemical synthesis of potential IP-DHA-a*t*RAL adducts

**Figure S8.** UPLC-MS chromatogram of the mixture of IP-DHA-a*t*RAL adducts synthesized chemically

**

**

**Figure S1.** Quantification of retinal, A2E, and full-field ERG in wild type and Abca4^-/-^ mice.

(**a**) 11-*cis* (11*c*RAL) and all-*trans* (a*t*RAL) retinal isomers were analyzed by normal phase HPLC from 7 month-old wild type (WT) and Abca4^-/-^ (KO) mice dark-adapted for 24 hours. The 11*c*RAL content significantly differs by Abca4 genotype. Error bars indicate SEM of the means (n = 12), *, p=0.034 unpaired t test (**b**) A2E quantification by reverse-phase HPLC from 3 month and 6 month- old wild type (WT) and Abca4^-/-^ (KO) mice. Abca4^-/-^ mice have increased accumulation of A2E relative to wild type animals. Quantification was performed from three age-matching mice. Representative chromatograms show absorbance at 430 nm. Peaks of A2E were identified thanks to an A2E synthetic standard. (**c**) Full-field (ff) ERG responses of wild type (WT) and Abca4^-/-^ (KO) between 2 and 8 months (WT 2-8 m and KO 2-8 m) or after 8 months of age (WT 9-12 m and KO 10-12 m). Scotopic ERG responses were recorded and a-wave amplitudes were plotted as a function of light intensity. ERG amplitudes were preserved in Abca4^-/-^ up to 8 months as compared to WT controls but declined approximately twice from 10 months of age. Error bars indicate SEM of the means (n ≥ 5). ** p=0.008 Mann Whitney test, KO 2-8 months vs.10-12 months.





**Figure S2.** Correlation between anatomical and functional evaluations

(**a**) Scatterplots for ONL/INL ratio and a-wave amplitude, and (**b**) scatterplots for 11*c*RAL content and a-wave amplitude, of individual treated *Abca4*^-/-^ mouse. The Pearson’s r correlation coefficients were 0.82 (a) and 0.85 (b), respectively. These two values indicate a linear correlation between the ONL/INL ratio and the a-wave (n=51) and 11*c*RAL rate and a-wave amplitudes with p<0.0001 (n=29).





**Figure S3.** Kinetics of retinoid in bleached Abca4^+/-^ mice

(**a**), Abca4^+/-^ mice (3 each point) were dark-adapted for 24 hours, the pupil dilated 15 min and exposed to bright light for different times as indicated. Retinoid were extracted from eyeballs in the presence of retinyl acetate to measure the extraction rate and 11*c*RAL, a*t*RAL and a*t*RPalm were quantified from standard calibration. The data are from a representative experiment and presented as means ± SD of triplicates relative to retinoid content (percentage of total). (**b**), normal-phase HPLC chromatogram of retinoid from a dark-adapted mouse showing UV absorption at 350 nm. Identified a*t*RPalm, retinyl acetate (Std), 11*c*RAL, and a*t*RAL are labeled.


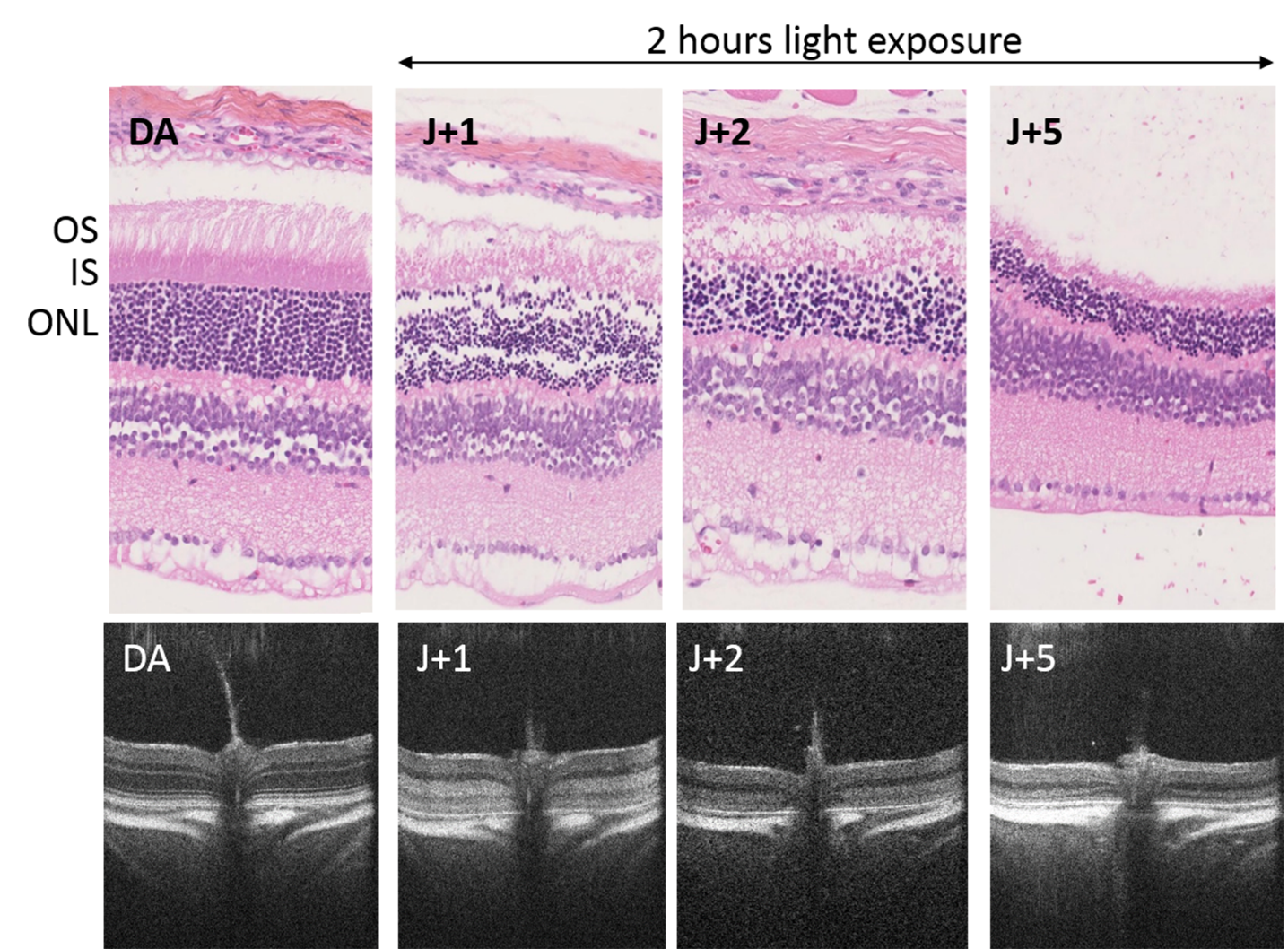


**Figure S4.** Kinetic of retinal damage in darkness after acute light exposure

Abca4^-/-^ mice adapted to darkness were exposed to bright light (20,000 lux, 2 hours) and kept in darkness for 1, 2 and 5 days before retinal morphology was analyzed on SD-OCT imaging (bottom) followed by hematoxylin/eosin/saffron stained (HES) eye cryosections by the Nanozoomer slide scanning system and NDP View Software (top). DA, mice adapted to darkness have an intact outer nuclear layer (ONL) with inner (IS) and outer (OS) segments. The outer nuclear layer has a hyporeflectivity with OCTs. After 1 day, the photoreceptor layers (outer nuclear and segments) are damaged. This corresponds to hyperreflectivity in OCT imaging. 2 days after light exposure, the photoreceptor nuclei layers are significantly reduced. The reflectivity of the OCT decreases slightly. After 5 days, the loss of photoreceptors rises and is correlated with a loss of reflectivity.


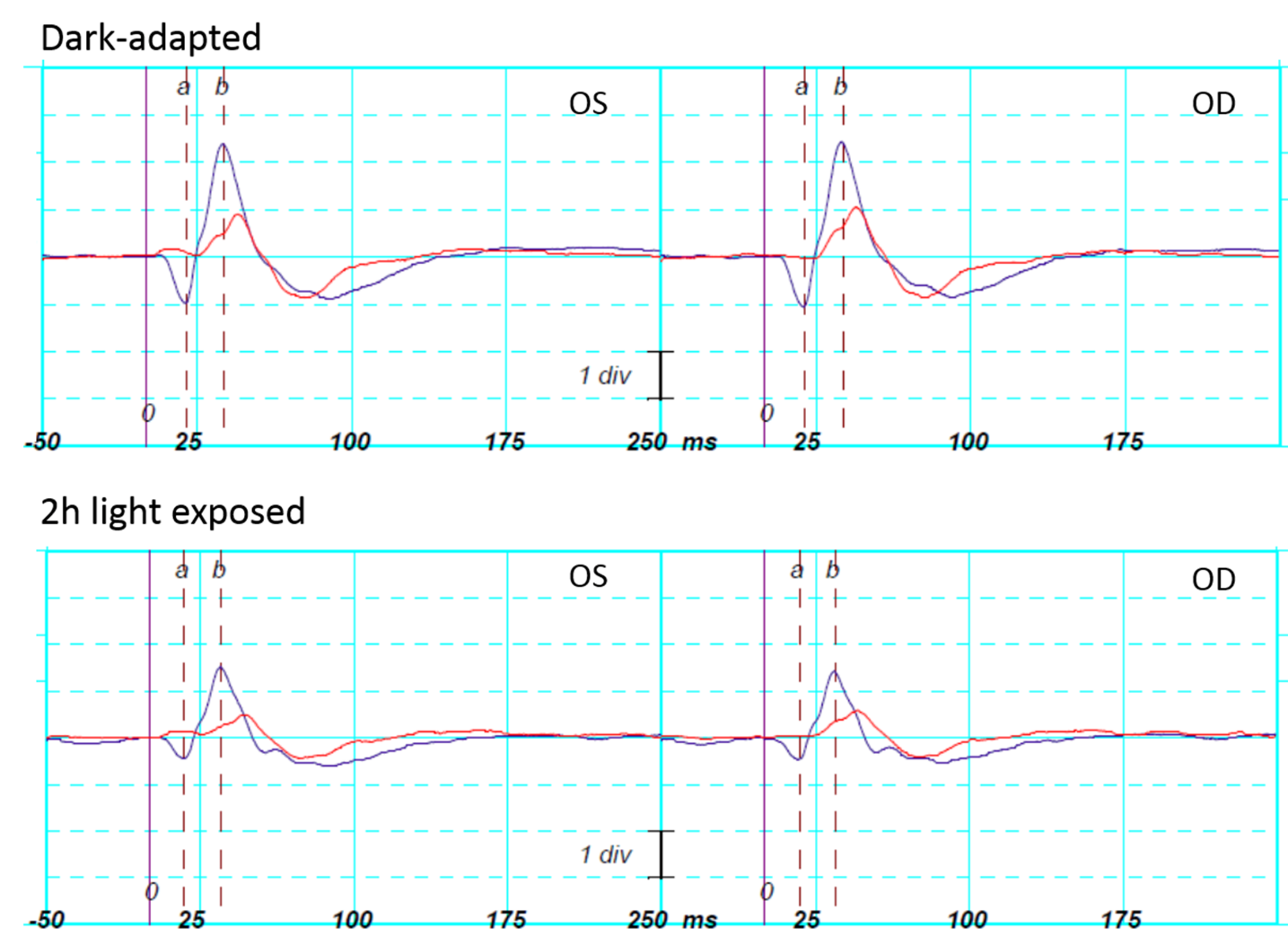


**Figure S5.** The light responses of rod and cone photoreceptors

Abca4^-/-^ mice were analyzed in scotopic ERG to measure rod + cone responses (purple lines, dark-adapted mice, OS (left eye), a =-152 µV, b = 526 µV; OD (right eye), a = -166 µV, b = 547 µV; 2h light exposed mice, OS, a = -69µV, b = 306µV, OD, a = -72µV, b = 295 µV), Photopic ERG for cone responses was recorded followed by 5 min bleaching at 2 log.cd.s.m^-2^ achromatic light intensity (red lines, dark-adapted mice, OS, b = 157 µV; OD, b = 133 µV; 2h light exposed mice, OS, b= 90µV, OD, b = 76µV)). Vertical dotted lines indicate the scotopic a- and b-waves. Amplitude scale, 1 div = 156µV. The mice were subjected to seven flashes of 2 log.cd.s.m^-2^ achromatic light intensity, amplitudes and latencies were averaged to quantify light responses. Mesopic and photopic responses were decreased by acute exposure to light, suggesting a loss of rods and cones in similar proportions.





**Figure S6.** Correlation between photoreceptor segment length, outer nuclear layer and photoresponse

**(a**), Scatterplots for photoreceptor segment (PS) length and ONL/INL ratio, and (**b**), scatterplots for PS length and a-wave amplitude, of individual treated Abca4 KO mouse. The Pearson’s r correlation coefficients were 0.954 (**a**) and 0.804 (**b**), respectively. These two values indicate a linear correlation between the PS length and the ONL/INL ratio, and between the PS length and the a-wave amplitude with p<0.0001 (n = 51).

**Figure S7.** Chemical synthesis of potential IP-DHA-a*t*RAL adducts

Reagents and conditions (Crauste et al. 2014): **(*i*)** acetic acid (1 eq), EtOH, rt, 27 h, 12%; **(*ii*)** DHA (1.1 eq), DCC (1.1 eq), DMAP (0.5 eq), CH_2_Cl_2_, rt, 2h, 47%. 2 major chromene adducts (1a and 1ba) were obtained by reaction of isopropyl-phloroglucinol (IP) with a*t*RAL in acidic conditions as described by Crauste et al. in 2014 and Cia et al. in 2016. 2 minor chromene derivatives (1’a and 1’b) probably having a different conformation of the cyclohexene ring (6s-*trans* instead of 6s-*cis*) are also present as it has been described for a*t*RAL by Wada et al. in 1998. The resulting mixture (the four adducts 1) was then esterified in Steglich conditions with DHA fatty acid producing 4 different adducts 2 named adduct 2ab and 2b for the major ones and 2’a and 2’b for the minor conformers.

Crauste C., C. Vigor, P. Brabet, M. Picq, M. Lagarde, C. Hamel, T. Durand and J. Vercauteren. 2014. Synthesis and evaluation of polyunsaturated fatty acid-phenol conjugates as anti-carbonyl-stress lipophenols. *Eur J Org Chem* 4548-4561.

Cia D., A. Cubizolle, C. Crauste, N. Jacquemot, L. Guillou, C. Vigor, C. Angebault, C. Hamel, J. Vercauteren and P. Brabet. 2016. Phloroglucinol protects retinal pigment epithelium and photoreceptor against all-trans-retinal induced toxicity and inhibits A2E formation. *J Cell Mol Med* 20(9): 1651-1663.

Wada A., A. Akai, T. Goshima, T. Takahashi and M. Ito. 1998. Determination of 6s.trans conformation of retinal chromophore in sensory rhodopsin I and photorhodopsin. *Bioorg Med Chem Lett* 8: 1365-1368.


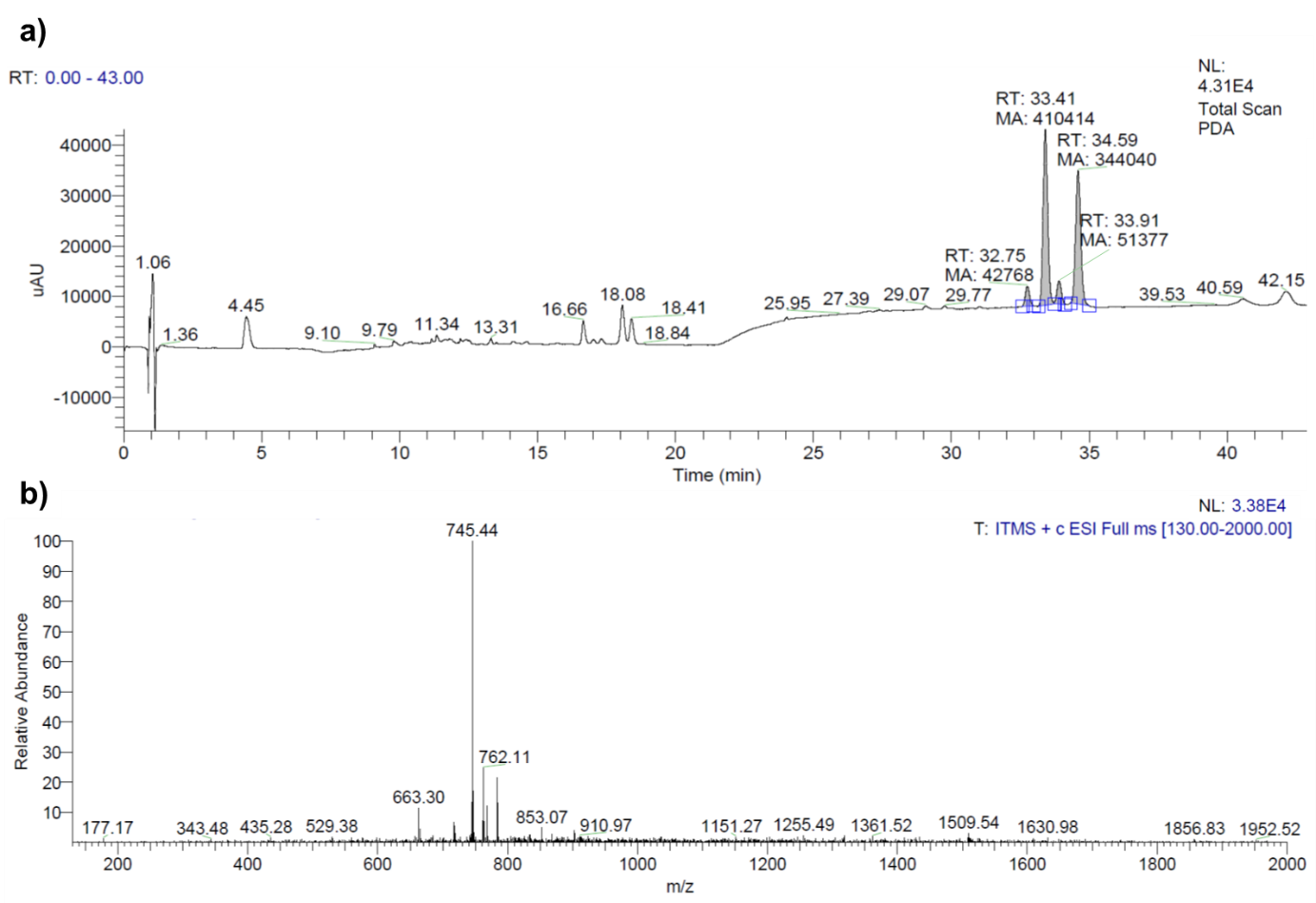


**Figure S8.** UPLC-MS chromatogram of the mixture of IP-DHA-a*t*RAL adducts synthesized chemically

**a)** Total scan PDA with evidence of the 4 different IP-DHA-a*t*RAL adducts (RT: 32.75; 33.41; 34.59 and 33.91) and **b)** ESI full MS spectrum of the 4 different adducts. UPLC-MS method: gradient of water + 0.1% formic acid / acetonitrile + 0.1% formic acid; t_0’_ = 80/20, t_5’_ = 80/20, t_10’_ = 13/87, t_20’_ = 13/87, t_21’_ = 0/100, t_43’_ = 0/100; flow = 300 µL/min; column Waters XBridge C18, 2.5 µM (2.1 x 100 mm) with XPVanGuard XBridge BEH C18, 2.5 µM (2.1 x 5 mm); MS (ESI+): [M + H]^+^ = 745.44.
